# The Diagnostic Yield of Cone-Beam Computed Tomography for Degenerative Changes of the Temporomandibular Joint in Dogs

**DOI:** 10.3389/fvets.2021.720641

**Published:** 2021-08-04

**Authors:** Rachel Marie McKay, Natalia Vapniarsky, David Hatcher, Nicole Carr, Shuai Chen, Frank J. M. Verstraete, Derek D. Cissell, Boaz Arzi

**Affiliations:** ^1^Department of Surgical and Radiological Sciences, School of Veterinary Medicine, University of California, Davis, Davis, CA, United States; ^2^Department of Pathology, Microbiology and Immunology, School of Veterinary Medicine, University of California, Davis, Davis, CA, United States; ^3^Department of Animal Sciences, University of California, Davis, Davis, CA, United States; ^4^Division of Biostatistics, Department of Public Health Sciences, University of California, Davis, Davis, CA, United States

**Keywords:** histology, osteoarthritis, degenerative joint disease, dogs, cone-beam CT, TMJ degeneration

## Abstract

Degenerative changes of the temporomandibular joint (DTMJ) may be diagnosed via cone - beam computed tomography (CBCT). However, despite advancement in CBCT imaging, correlation of DTMJ features identified on CBCT with gross and histological findings is currently limited. This study aimed to correlate CBCT findings of DTMJ of dogs with gross and histopathologic changes. Temporomandibular joints (TMJ) (*n* = 38) from fresh cadaver heads of asymptomatic dogs (*n* = 19) were examined radiologically, macroscopically, and microscopically. Association of CBCT - detected DTMJ changes with gross and histological findings were statistically evaluated via kappa statistics and ordinal logistic mixed-effects models. The radiological changes observed on CBCT included joint space narrowing, subchondral/cortical bone changes (i.e., erosions or lysis), osteophytes, and subchondral bone sclerosis. Upon macroscopic evaluation, the majority of examined specimens had mild changes with cartilage defects and osteophytes affecting <10% of the total articular surface area. Histopathologic changes comprised splitting and degeneration of the fibrous cartilage layers, subchondral bone exposure, subchondral bone sclerosis, focal subchondral bone lysis, and occasional cell death. Subchondral sclerosis was the most prevalent finding radiologically and histologically with a fair to excellent agreement. Importantly, the more severe the TMJ degenerative changes, the higher the agreement between CBCT and histology. Based on the correlative results of statistical analysis, CBCT was found to be a suitable modality to evaluate DTMJ.

## Introduction

The temporomandibular joint (TMJ) is a unique synovial joint found in mammalian species consisting of the mandibular head and the mandibular fossa (1). Also, the joint has an intra-articular fibrocartilaginous disc dividing the joint into two non-communicating compartments (2–4). From a kinematic perspective, the TMJ of dogs is capable of a hinge-like motion (i.e., opening and closing) with limited lateral movement (3, 5). In contrast, the TMJ of humans is capable of more diverse kinematics such as opening and closing, rotation, translation, protrusion, retrusion, and laterotrusion (2, 6, 7). Regardless, it is important to note that the mandibles are the only bones that move via the TMJ, while the mandibular fossa remains stationary (1, 7). Furthermore, the anatomical features of the TMJ directly correlate with its function as a load-bearing joint, in contrast to the weight-bearing appendicular joints (8). Specifically, the articulating surfaces of the TMJ are covered by fibrocartilage, while the articulating surfaces of appendicular synovial joints are covered by hyaline cartilage (4, 9, 10). Fibrocartilage is unique as it contains both types I and II collagen, whereas hyaline articular cartilage mainly contains type II collagen (10, 11). Due to its fibrous structure, fibrocartilage withstands shear and tension forces better than hyaline cartilage and is, therefore, better suited to endure the gliding motion experienced by the TMJ (10, 12).

Degenerative changes of the TMJ are commonly diagnosed in humans, and the TMJ disc may play a significant role in its pathophysiology by contributing to clinical signs such as pain, joint noise during use (i.e., clicking), and locking of the joint (13–16). Similarly, naturally occurring DTMJ is also being increasingly recognized in animals and is the most common TMJ disorder identified in dogs (3, 17, 18). In addition, TMJ disc perforation has been recently identified in dogs with TMJ pathology (3). However, neither the role of the disc in TMJ disorders nor the extent of the pathology underlying these abnormalities of the TMJ of dogs are fully understood.

Despite advancement in CBCT imaging, the understanding of the correlation of DTMJ features identified on CBCT with gross and histological findings in both human and veterinary medicine is currently limited. Past reports have focused on comparing imaging findings with joint pathology and/or osteoarthritis in appendicular joints (19–22). In addition, a recently published review outlined 21 studies in human patients that have correlated histological features to subchondral bone abnormalities (SBAs) found on magnetic resonance imaging in patients with various musculoskeletal disorders, including osteoarthritis, rheumatoid arthritis, spondyloarthritis, and degenerative disc disease (21). Though these studies suggest that a correlation exists between SBAs and certain histological features, most studies did not meet the quality criteria and thus, further research that focuses on quality and consistency is needed (21). Studies in humans comparing computed tomography (CT) abnormalities with histopathological findings on non-TMJ disorders such as periprosthetic defect healing, otosclerosis, and the grading of pulmonary nodules and peripheral lung adenocarcinoma have found a positive correlation between imaging and histopathology (23–26).

Therefore, to improve the diagnostic accuracy of DTMJ, this study sought to correlate CBCT findings of DTMJ in dogs with the definitive characterization of the disorder by gross and histopathologic evaluation. We hypothesized that CBCT would significantly correlate with gross and histological findings for the presence and severity of DTMJ.

## Materials and Methods

### Specimen Acquisition

The TMJs from 19 skeletally mature domestic dogs were obtained from dogs admitted to the Veterinary Medical Teaching Hospital, University of California, Davis; all dogs were humanely euthanized for reasons unrelated to the study and had owner consent for research or unrestricted use. The dogs were from the following breeds: Labrador retriever (*n* = 3), Australian cattle (*n* = 1), mixed breed (*n* = 11), Airedale terrier (*n* = 1), German shepherd (*n* = 1), border collie (*n* = 1), and English Springer spaniel (*n* = 1). The specimens consisted of six castrated males and 10 spayed females (three with unknown gender) ranging from 3 to 16 years of age, median of 11-years-old (three with unknown age). All specimens were obtained fresh (post-mortem interval <24 h) and kept frozen at −20°C until dissection and analysis. All specimens were thawed at 4°C prior to use and evaluation. None of the 19 dog's medical records indicated clinical signs associated with potential TMJ pathology.

### Cone-Beam CT Imaging

CBCT (NewTom 5G, Verona, Italy) was used to obtain high-resolution scans of both TMJs for each dog. The scans were performed using a 16 × 12 cm field of view, 24 s scan time and a 0.2 mm isotropic voxel size. The primary reconstruction created a voxel volume that could be visualized in slices as thin as 0.2 mm and oriented in any orthogonal, oblique or curved plane. The CBCT parameters and scan time are identical to the those used for clinical cases in our institution and are done under general anesthesia or very heavy sedation. Each skull was scanned twice in sternal recumbency: one in an open-mouth position, which was obtained with the jaws opened at 45°, and one in a closed-mouth position.

Images were examined on medical-grade flat-screen monitors (ASUS PB278Q, ASUSTeK Computer Inc.,Taipai, Taiwan) using commercially available specialized software (Anatomage Invivo5; Anatomage Inc., Santa Clara, California). Prior to visualization and evaluation, each scan was re-oriented and followed by a Cartesian coordinate transformation. The re-orientation was performed with 6 degrees of freedom (X, Y, Z, Yaw, Pitch, and Roll). Yaw was adjusted in the axial view using two points on the skull base where the zygomatic arch connects with the squamous part of the temporal bone. Pitch was adjusted in the sagittal view level with the palatal plane, and Roll was adjusted in coronal view to level the ventral orbital rims. The medial and lateral pole of the condylar process of each TMJ was then defined, and serial sections were taken both parallel and perpendicular to that axis. The images were evaluated blindly by a oral and maxillofacial radiologist (DCH) experienced in assessing the TMJ in humans and animals. All images were evaluated in two opposing planes. CBCT images of the TMJ were scored for degenerative changes, according to the 4-point semiquantitative system defined in our previous imaging study (Table 1) (17). The medial, central, and lateral regions of the mandibular head and fossa were evaluated for the presence of irregularities, including subchondral bone integrity, sclerosis, cystic lesions, altered morphology, and osteophytes. Additionally, the medial, central, and lateral aspects of each joint space were assessed for narrowing and any abnormalities suggestive of the TMJ disc changes were recorded. The severity of osteoarthritis in each mandibular fossa and the mandibular head was graded separately, and then an overall grade was assigned to each TMJ according to the system in Table 1 (17).

**Table 1:** CBCT Grading system for severity of DTMJ of the temporomandibular joint (17).

**Grades**	**Description**
0	No DJD detected
1	Mild (early signs of periarticular new bone formation with minimal or no joint space narrowing or subchondral bone change)
2	Moderate (moderate periarticular new bone formation, joint space narrowing, or subchondral bone sclerosis)
3	Marked (severe periarticular new bone formation, joint space narrowing, or subchondral bone sclerosis or lysis)

### Gross and Histological Evaluation

Following imaging, the mandibular head and fossa of both TMJs were dissected and evaluated grossly by a board-certified veterinary pathologist (NV) according to the International Cartilage Repair Society and Osteoarthritis Research Society International (ICRS/ORSI)-established scheme for appendicular joints since no such system was established or fully validated for the TMJ (27). High-resolution digital photographs of the samples were obtained with Nikon digital camera with an 18–55 mm 1:3.5–5.6G zoom lens mounted on a stand. Imaging analysis software (ImageJ, National Institutes of Health, Bethesda, MD) was used to quantify the total area of each articular surface and the areas where pathology was evident. The surface area of cartilage defects (i.e., erosion or defects of the articular surface, thickening or roughness) and osteophytes was expressed as a percentage of the total articular surface of that joint (mandibular fossa or mandibular head). According to the extent of surface area abnormalities, a DTMJ grade was assigned from 0 to 4 (0: no abnormalities, 1: <10%, 2: 10–25%, 3: 25–50%, 4: >50%). TMJ discs were also collected during these initial joint dissections and evaluated grossly for overall shape, integrity, evidence of thinning, or perforation.

After gross evaluation, the mandibular head and fossa of each TMJ and the TMJ disc were fixed in 10% buffered formalin. The osseous components were then decalcified in 10% formic acid for 3–4 weeks and subsequently sectioned sagitally. The rostral and caudal sections were then embedded in paraffin. Five μm sections were cut from each block at 200 μm intervals, mounted, and stained with hematoxylin & eosin (H&E) according to standard protocol (28). On average, 6 slides were evaluated histologically per each condylar process and fossa, thus creating 12 slides per TMJ or 24 slides per dog. Each articulating surface was histologically assessed and assigned an osteoarthritis (OA) stage from 0 to 6 (Table 2) (27). A total joint OA score was calculated by multiplying the DTMJ stage by the DTMJ grade for each mandibular head and fossa (27).

**Table 2:** Staging system for histologic evaluation of the mandibular head and fossa of the TMJ (27).

**Stage**	**Description**
0	Surface intact, all cells viable
1	Superficial fibrillation; mild alteration in cell orientation, density or distribution
2	Deeper fibrillation; superficial cell death or proliferation; alteration in extracellular matrix density
3	Deep fibrillation; more extensive cell death or proliferation
4	Complete loss of articular cartilage with subchondral bone exposure
5	Loss of articular cartilage plus subchondral bone sclerosis
6	Remodeling of the articular bones, deformation of the articulating surface, osteophyte formation, subchondral cysts, and/or bone marrow inflammation

Following fixation in formalin, the TMJ discs were cut in the rostrocaudal (antheroposterior) direction onto 4–5 ribbons, embedded in paraffin, sectioned at 5 μm, and stained with H&E. The samples were then assessed histologically.

### Statistical Analysis

Frequencies and proportions of assessment in mandibular head and fossa were reported separately for left and right sides. There were a few missing values in some measurements due to the inability to assess some samples (missing proportion was within 0–25%), and those missing values were excluded in all analyses.

Kappa (κ) statistic was used as a measure of agreement between two assessment methods. A κ statistic in the range of 0.81–1.00 was interpreted as excellent, 0.61–0.80 as substantial, 0.41–0.60 as moderate, 0.21–0.40 as fair, 0.01–0.20 as slight, and ≤0.00 as poor agreement. We calculated the point estimate of κ for binary assessment (yes/no), unbiasedly ignoring the correlation within the cluster (29). When comparing joint space narrowing by CBCT (measured for joint) with cartilage damage by histology (measured for mandibular head and fossa separately), we defined cartilage damage as “yes” for the joint if either mandibular head or fossa was “yes.”

To evaluate the association between radiological OA Grade (CBCT) and combined gross and histological OA score (OA Score = OA stage ^*^ OA grade), we used ordinal logistic mixed-effects models. We reported odds ratios on increasing to the next higher level of outcome, where odds ratio>1 indicates a higher probability to a higher grade of the outcome. Fixed effects included CBCT OA Grade (categorized as low = 0/1 or high = 2/3), side (left or right), and the interaction between them. The interactions were removed from the final model due to non-significance. A random intercept was included in the model to account for within-animal correlation whenever necessary. The second set of models was also fitted, which did not adjust for sides.

Data were analyzed using R 3.6.1 (R Foundation for Statistical Computing, Vienna, Austria) for κ statistic and SAS version 9.4 (SAS Institute, Cary, NC) for descriptive statistics and ordinal logistic mixed-effects models. A two-sided *p* < 0.05 was considered statistically significant.

## Results

### CBCT Findings

The TMJ changes observed on CBCT included periarticular new bone formation, joint space narrowing, subchondral bone sclerosis, and subchondral/cortical bone erosions or lysis. Bone sclerotic changes were the most common findings and noted in 33 out of the 38 mandibular heads (86.8%) and 36 out of the 38 (94.7%) of the mandibular fossae.

#### Joint Space Narrowing Was Often Present in Conjunction With Subchondral Bone Sclerosis

**Figure 1:**
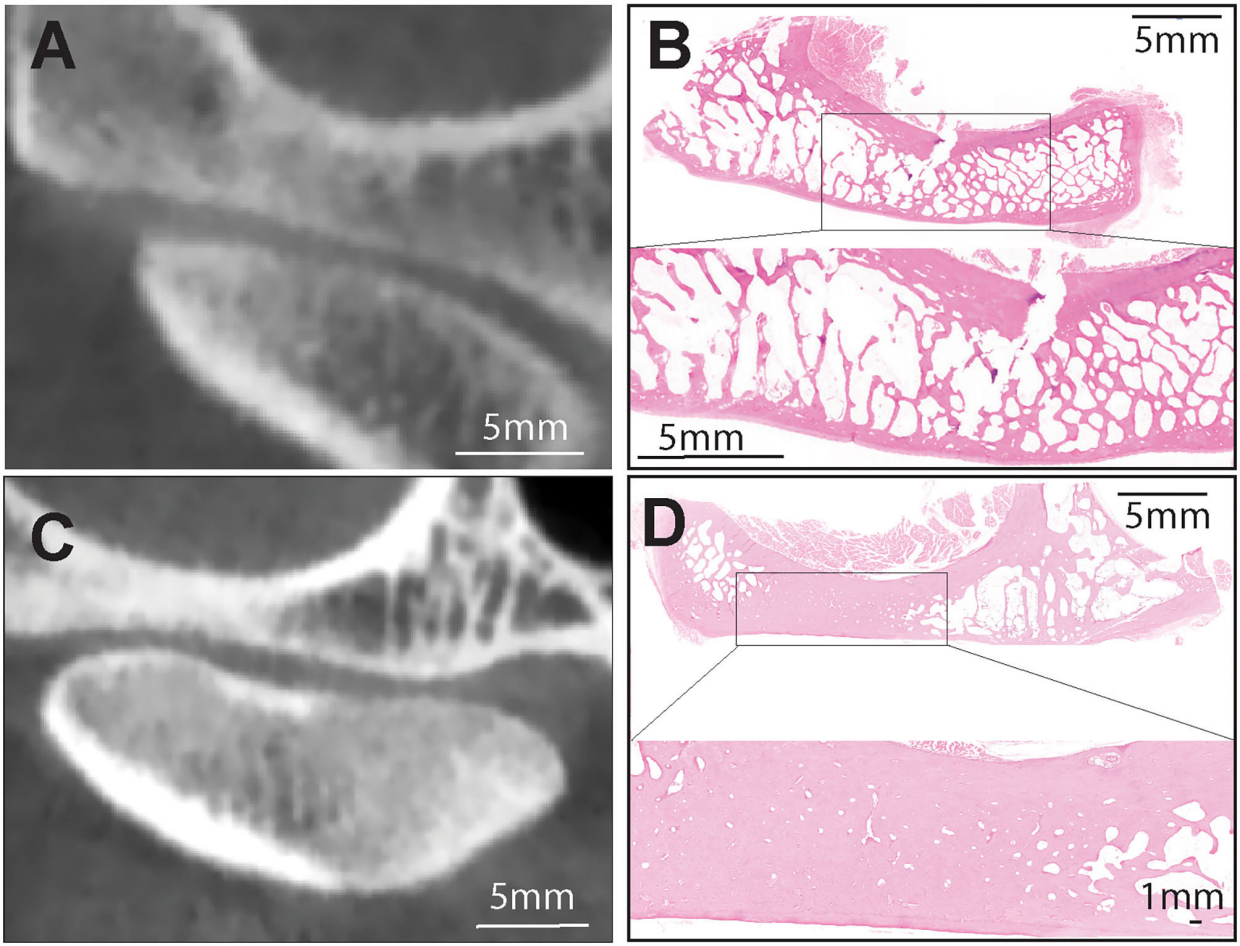
**(A,B)** Representative transverse CBCT and histology images of a dog demonstrating normal, non-sclerotic bone of mandibular fossa and condylar process. **(C)** Representative transverse CBCT image of a dog demonstrating joint space narrowing and subchondral bone sclerosis at the mandibular fossa which correlated with excellent agreement with histologic evidence of sclerosis **(D)**. H&E staining of the mandibular fossa depicted in **(C)**. Note extensive area of bone sclerosis characterized by reduction of marrow cavities as compared to other areas in the mandibular fossa. Bar = 1 mm and closeup Bar = 5 mm.

In 31 out of the 38 (81.6%) of the TMJs evaluated, there were various degrees of joint space narrowing (Table 3; Figure 1). The majority of both subchondral bone sclerosis and joint space narrowing were located on the lateral aspects of the joints. However, 7 of the 31 (22.6%) TMJs with narrowed joint spaces did not have lateral involvement. Similarly, 4 of the 33 (12.1%) mandibular heads and 4 of the 36 (11.1%) mandibular fossae with sclerotic changes had sclerosis localized medially and/or centrally (no lateral involvement).

**Table 3:** Summary of frequency and proportion of assessment.

	**Left**		**Right**		**Both Sides**	
	**(** ***N*** **= 19)**		**(** ***N*** **= 19)**		**(** ***N*** **= 38)**	
	**Head**	**Fossa**	**Head**	**Fossa**	**Head**	**Fossa**
	***N* (%)**	***N* (%)**	***N* (%)**	***N* (%)**	***N* (%)**	***N* (%)**
**Sclerosis (RADIOLOGICAL)**						
No	2 (10.5%)	1 (5.3%)	3 (15.8%)	1 (5.3%)	5 (13.2%)	2 (5.3%)
Yes	17 (89.5%)	18 (94.7%)	16 (89.5%)	18 (94.7%)	33 (86.8%)	36 (94.7%)
**Sclerosis (HISTO)**						
No	5 (35.7%)	1 (5.9%)	5 (33.3%)	0 (0.0%)	10 (34.5%)	1 (3.2%)
Yes	9 (64.3%)	16 (94.1%)	10 (66.7%)	14 (100.0%)	19 (65.5%)	30 (96.8%)
**Osteophytes (RADIOLOGICAL)**						
No	11 (57.9%)	19 (100.0%)	16 (84.2%)	18 (94.7%)	27 (71.1%)	37 (97.4%)
Yes	8 (42.1%)	0 (0.0%)	3 (15.8%)	1 (5.3%)	11 (28.9%)	1 (2.6%)
**Osteophytes (HISTO)**						
No	7 (46.7%)	17 (100.0%)	13 (86.7%)	13 (92.9%)	20 (66.7%)	30 (96.8%)
Yes	8 (53.3%)	0 (0.0%)	2 (13.3%)	1 (7.1%)	10 (33.3%)	1 (3.2%)
**SCB lysis/cysts (RADIOLOGICAL)**						
No	13 (68.4%)	14 (73.7%)	15 (78.9%)	13 (68.4%)	28 (73.7%)	27 (71.1%)
Yes	6 (31.6%)	5 (26.3%)	4 (21.1%)	6 (31.6%)	10 (26.3%)	11 (28.9%)
**SCB lysis/cysts (HISTO)**						
No	9 (69.2%)	14 (93.3%)	13 (86.7%)	14 (100.0%)	22 (78.6%)	28 (96.6%)
Yes	4 (30.8%)	1 (6.7%)	2 (13.3%)	0 (0.0%)	6 (21.4%)	1 (3.4%)
**Cartilage damage (HISTO)**						
No	7 (50.0%)	10 (71.4%)	9 (60.0%)	9 (64.3%)	16 (55.2%)	19 (67.9%)
Yes	7 (50.0%)	4 (28.6%)	6 (40.0%)	5 (35.7%)	13 (44.8%)	9 (32.1%)
**OA Grade (GROSS)**						
0	3 (17.7%)	7 (43.8%)	2 (12.5%)	4 (23.5%)	5 (15.2%)	11 (33.3%)
1	6 (35.3%)	6 (37.5%)	3 (18.9%)	9 (52.9%)	9 (27.3%)	15 (45.5%)
2	3 (17.7%)	1 (6.3%)	6 (37.5%)	3 (17.7%)	9 (27.3%)	4 (12.1%)
3	5 (29.4%)	1 (6.3%)	5 (31.3%)	1 (5.9%)	10 (30.3%)	2 (6.1%)
4	0 (0.0%)	1 (6.3%)	0 (0.0%)	0 (0.0%)	0 (0.0%)	1 (3.0%)
**OA Stage (HISTO)**						
1	2 (13.3%)	0 (0.0%)	3 (20.0%)	0 (0.0%)	5 (16.7%)	0 (0.0%)
2	0 (0.0%)	0 (0.0%)	1 (6.7%)	0 (0.0%)	1 (3.3%)	0 (0.0%)
3	2 (13.3%)	1 (5.9%)	0 (0.0%)	0 (0.0%)	2 (6.7%)	1 (3.2%)
5	2 (13.3%)	15 (88.2%)	8 (53.3%)	13 (92.9%)	10 (33.3%)	28 (90.3%)
6	9 (60.0%)	1 (5.9%)	3 (20.0%)	1 (7.1%)	12 (40.0%)	2 (6.5%)
TOTAL Path OA Score (OA stage * OA grade)						
0	3 (20.0%)	7 (46.7%)	2 (14.3%)	3 (21.4%)	5 (17.2%)	10 (34.5%)
1	0 (0.0%)	0 (0.0%)	1 (7.1%)	0 (0.0%)	1 (3.5%)	0 (0.0%)
2	0 (0.0%)	0 (0.0%)	2 (14.3%)	0 (0.0%)	2 (6.9%)	0 (0.0%)
3	1 (6.7%)	0 (0.0%)	0 (0.0%)	0 (0.0%)	1 (3.5%)	0 (0.0%)
5	2 (13.3%)	4 (26.7%)	1 (7.1%)	7 (50.0%)	3 (10.3%)	11 (37.9%)
6	4 (26.7%)	1 (6.7%)	0 (0.0%)	1 (7.1%)	4 (13.8%)	2 (6.9%)
10	0 (0.0%)	1 (6.7%)	3 (21.4%)	2 (14.3%)	3 (10.3%)	3 (10.3%)
12	2 (13.3%)	0 (0.0%)	1 (7.1%)	0 (0.0%)	3 (10.3%)	0 (0.0%)
15	0 (0.0%)	1 (6.7%)	2 (14.3%)	1 (7.1%)	2 (6.9%)	2 (6.9%)
18	3 (20.0%)	0 (0.0%)	2 (14.3%)	0 (0.0%)	5 (17.2%)	0 (0.0%)
20	0 (0.0%)	1 (6.7%)	0 (0.0%)	0 (0.0%)	0 (0.0%)	1 (3.5%)
**OA Grade (CBCT)**						
0	0 (0.0%)	1 (5.6%)	0 (0.0%)	0 (0.0%)	0 (0.0%)	1 (2.8%)
1	4 (22.2%)	2 (11.1%)	6 (33.3%)	4 (22.2%)	10 (27.8%)	6 (16.7%)
2	9 (50.0%)	13 (72.2%)	9 (50.0%)	11 (61.1%)	18 (50.0%)	24 (66.7%)
3	5 (27.8%)	2 (11.1%)	3 (16.7%)	3 (16.7%)	8 (22.2%)	5 (13.9%)
	**Left Joint**		**Right Joint**		**Both Joints**	
	**(** ***N*** **= 19)**		**(** ***N*** **= 19)**		**(** ***N*** **= 38)**	
**Joint space narrowing (RADIOLOGICAL)**						
No	4 (21.1%)		3 (15.8%)		7 (18.4%)	
Yes	15 (78.9%)		16 (84.2%)		31 (81.6%)	

In 29 of the 38 (76.3%) joints, sclerosis was found on the aspect of at least one of the articulating surfaces that directly opposed the areas of most significant joint space narrowing. However, only 3 of these 29 joints (10.3%) had identical localizations of sclerosis between both mandibular head and fossa, and in conjunction with joint space narrowing. In the 26 other joints, sclerosis was also found in areas where joint space narrowing was not observed, and sclerosis was not specifically observed on both articulating surfaces in these instances.

Sclerosis was noted on one or both articulating surfaces in 30 of the 31 joints (96.8%) that had identifiable joint space narrowing. One joint out of the 38 TMJs did not exhibit this pattern, where joint space narrowing was observed without sclerosis. On the contrary, if sclerosis was observed, there was not necessarily joint space narrowing. In 7 out of the 38 joints (18.4%), sclerosis was observed on both articulating surfaces, but joint space narrowing was not observed upon evaluation of the CBCT images.

#### Erosive Subchondral Bone Changes and Osteophytes Were Observed With Less Frequency Than Sclerosis

**Figure 2:**
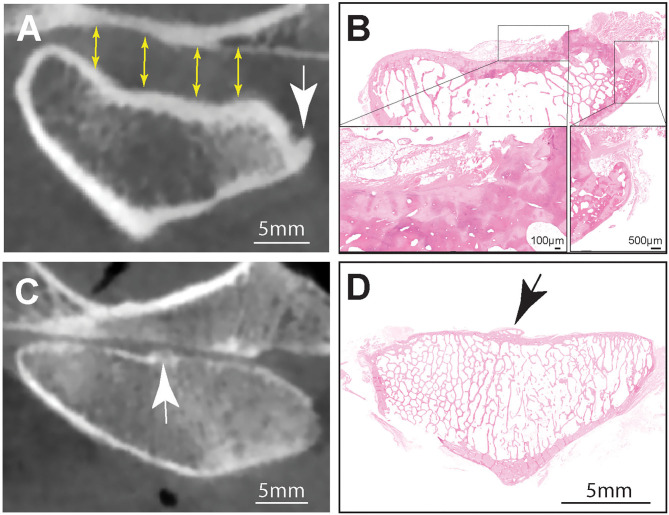
**(A)** Representative transverse CBCT image of the TMJ of a dog exhibiting increased joint space (yellow arrows), subcondral bone erosion and an osteophyte at the medial aspect of the mandibular head (white arrow). Note that there was excellent agreement between osteophytes found on CBCT and histology **(B)**. Hematoxilin & Eosin staining of the entire mandibular head shown in **(A)**. Note surface cartilage damage and large laterally located osteophyte. Inset (right) higher magnification of the osteophyte. Bar = 500 μm, Inset (left) Higher magnification of the sclerotic subchondral bone denuded from surface cartilge. The defect is overlayed by loose fibrous connective and adipose tissue. **(C)** A CBCT image of an osteophte present on the articular surface of the mandibular head (white arrow) of a dog had excellent agreement with histology (black arrow) **(D)**. Hematoxilin & Eosin staining of the entire mandibular head. Note centrally located osteophyte (black arrow), Bar = 5 mm.

**Figure 3:**
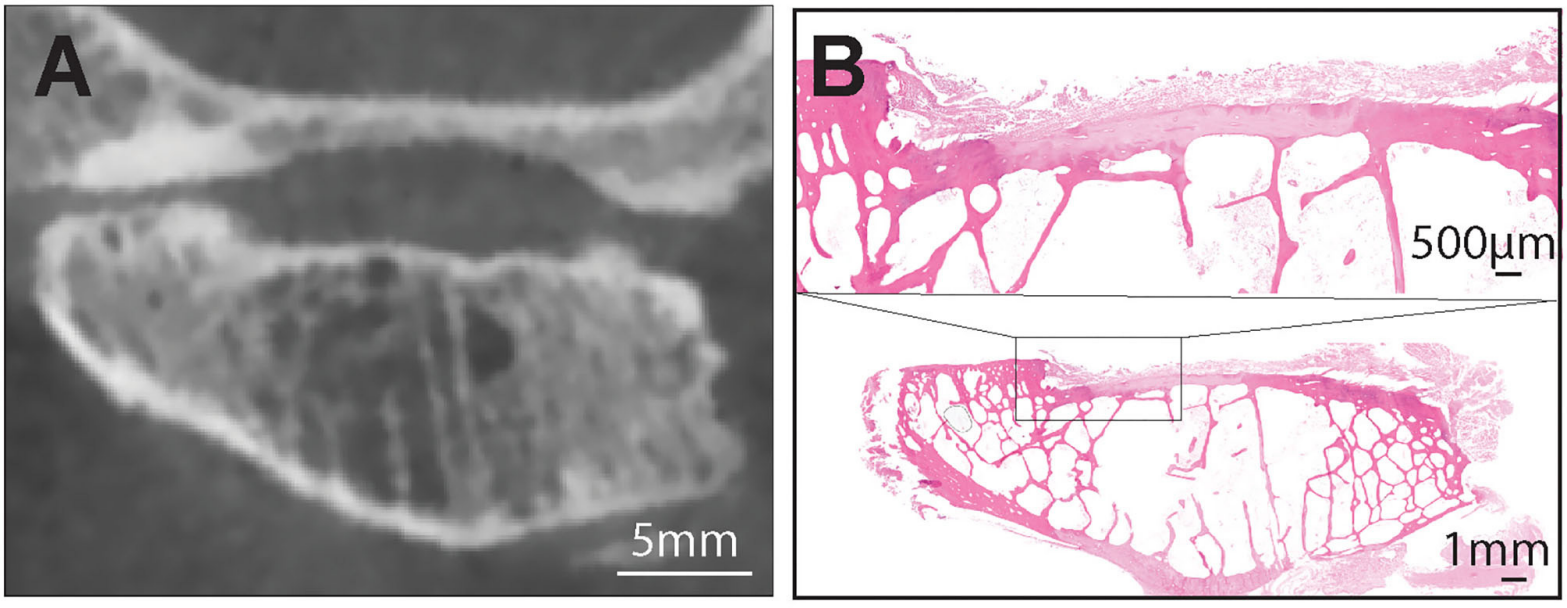
**(A)** A representative transverse CBCT image of the TMJ of the dog demonstrating joint space alterations and severe articular surface damage at the mandibular head. These alterations included subchondral bone defect, articular cartilage damage and subchondral bone cyst. Note that there was moderate agreement between histologic and radiologic findings of subchondral bone damage and cysts on the mandibular heads **(B)**. Hematoxilin & Eosin staining of the entire mandibular head with extensive damage of the cartilge and subchondral bone. Note large marrow cavities that could have been misinterpreted on CBCT as subchondral cysts. Bar = 1 mm. Inset: the exposed subchondral bone is covered by loose fibrous connective tissue. There is no evidence of bone sclerosis despite the extensive damage to cartilge. Bar = 500 μm.

10 out of the 38 (26.3%) mandibular heads and 11 of the 38 (28.9%) mandibular fossae had evidence of bone change, including erosions (*n* = 18), cortical defects (*n* = 10), and areas of subchondral bone lysis (cysts) (*n* = 4) (Table 3; Figures 2, 3). Though the distribution of such changes varied amongst the aspects of the articulating surfaces, nearly half of the changes noted on the mandibular heads and fossas had lateral localization (or were localized on the lateral aspect of the joint).

Additionally, cortical bone flattening or thinning was observed on 17.1% of surfaces (13 of 76), but these changes were attributed to normal breed variation in the shape of the TMJ (30). An osteophyte or enthesophyte was observed in 11 out of the 38 (28.9%) mandibular heads (Table 3; Figure 2). Only one of 38 mandibular fossas (2.6%) had an appreciable osteophyte on CBCT (Table 3).

#### The Majority of Joint Surfaces Were Assigned a Radiographic OA Grade 2

As described in Table 1, 18 of 36 (50%) mandibular heads and 24 of 36 (66.7%) mandibular fossas received an OA grade of 2 (Table 3). The remaining scores were distributed rather evenly amongst scores of 3 and 1 (Table 3). Only 1 mandibular fossa (2.6%) was assigned an OA grade 0. Additionally, in 25 out of 38 joints (69.4%), both mandibular heads and fossas received the same OA grade (Table 3).

### Gross Pathology Findings

#### The Majority of Mandibular Heads and Fossas Were Assigned a Gross OA Grade of 1

In 27.3% (9 of 33) of the mandibular heads and 45.5% (15 of 33) of mandibular fossas, <10% of the articular surface had surface abnormalities that were assigned a DJD grade 1 (Table 3). These surface abnormalities were represented by either erosion of the articular surface, evidence of thickening or roughness with or without discoloration. Of note, discoloration changes were disregarded in the absence of other changes of other surface abnormalities. The latter is due to known artifactual effects of freeze-thaw and post-mortem blood pooling. 15.2% (5 of 33) of mandibular heads and 33.3% (11 of 33) of mandibular fossas had no gross evidence of disease (DJD grade 0) (Table 3). The remaining scores were distributed rather evenly amongst grades of 2 and 3. Only 1 joint surface was assigned a gross DJD grade of 4, where >50% of the gross surface had evidence of disease (Table 3). Presumed osteophytes were noted on 5 of 70 surfaces (7.1%).

### Histopathology Evaluation

The microscopic changes included splitting and separation of fibers in the superficial layer of the fibrocartilage (fibrillation), thinning and complete loss of the fibrocartilage, subchondral bone sclerosis, osteophytes, subchondral bone lysis with prolapse of the fibrocartilage (cysts) and reduction of cellularity.

#### Subchondral Bone Sclerosis (SCB) Was the Most Common Histological Finding

Subchondral sclerotic changes were more common in the mandibular fossa, as opposed to the mandibular head. Sclerotic changes were found in 65.5% of evaluable mandibular heads and 96.8% of mandibular fossas (Table 3).

Findings characterized as “surface cartilage damage” included loss of articular cartilage with subchondral bone exposure, splitting and fibrillation of the fibrous cartilage layer, and cell loss represented by presence of acellular areas within the fibrocartilage matrix. Such changes were found in 44.8% of mandibular heads and 32.1% of mandibular fossas (Table 3).

#### Osteophytes, Subchondral Bone Lysis, and Subchondral Cysts Were More Commonly Found in the Mandibular Head

Subchondral bone lysis, cysts, and osteophytes were found with less frequency than sclerosis and cartilage damage on temporomandibular joint surfaces. Additionally, both features were more commonly found on the mandibular head. Osteophytes were noted in 33.3% of mandibular heads (10 of 30) and 3.2% of mandibular fossas (1 of 31) (Table 3). Following the trend of osteophytes, subchondral bone lysis and/or cysts were found in 21.4% (6 of 28) mandibular heads and 3.4% (1 of 29) mandibular fossas (Table 3).

Of note, osteophytes were difficult to discern from individual shape variability and shape changes, especially if located on the lateral or medial aspects of the mandibular head or the fossa. Additionally, fibrocartilage damage was difficult to discern from artifactual folding and separation of the tissue on the sections. In such cases, presence of neovascularization or decreased cellularity aided in diagnoses of fibrocartilage damage. Joint space narrowing was impossible to diagnose histologically because mandibular heads and fossas were evaluated after dissection and disarticulation.

#### High Histological Stages Were Commonly Assigned

In accordance with the grading system in Table 2, 90.3% of mandibular fossas (28 of 31) were assigned a stage 5 upon histological evaluation (Table 3) primarily due to evidence of sclerosis. Similarly, 33.3% of mandibular heads (10 of 30) were assigned a stage 5, and 40.0% (12 of 30) a stage 6 (Table 3). The defining feature of an OA stage of 5 is sclerosis. Thus, any surface that had any sclerosis was automatically assigned a grade 5. No joint surfaces were assigned an OA Grade of 4. The remaining joint surfaces were assigned lower OA Grades (Table 3).

#### Disc Perforation Naturally Occurs in Dogs

**Figure 4:**
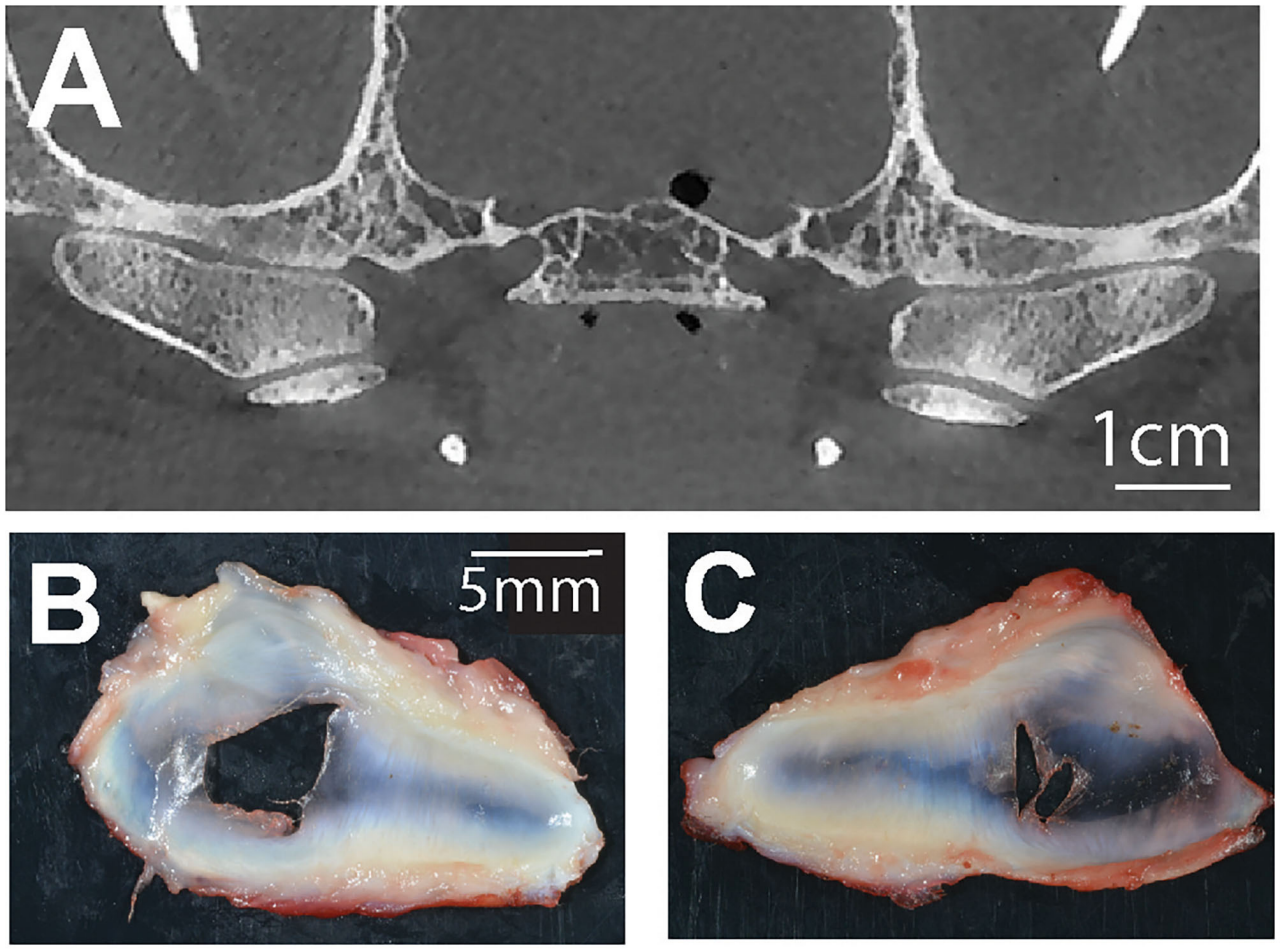
**(A)** A representative transverse CBCT image demonstrating bilateral joint space narrowing at the TMJ of a dog with corresponding bilateral TMJ disc perforations **(B,C)**.

In two cases, bilateral disc perforation was confirmed grossly (Figure 4). Microscopic examination of these discs revealed decreased cellularity of the fibrocartilage tissue especially in the vicinity of the perforation, separation and splitting of the collagen fiber bundles. Subjectively, they extend to where the synovial cells extended toward the central portions of the disc were increased in these cases (synovialization). The gross appearance and histomorphology of the remaining discs was within the reported normal (15).

## Agreements and Correlations

### Statistical Correlation Between Histopathology and CBCT

Four main categories were chosen as areas of focus for evaluation of correlation between the modalities of CBCT and histopathology: subchondral bone sclerosis, osteophytes, subchondral lysis/cysts, and joint space narrowing/surface cartilage damage. CBCT observation of joint space narrowing was correlated with histopathologic evidence of surface cartilage damage on either the fossa or the mandibular head. Kappa statistic (κ) measured the association between radiologic and histopathologic findings of each joint (Table 4). The association between OA grades assigned via CBCT, histopathologic OA score was evaluated via the use of ordinal logistic mixed-effects models and reported odds ratios (Table 5).

**Table 4:** Summary of kappa statistics for agreement of binary assessment (yes/no).

	**κ^a^**	
**Compared Pair**	**Head**	**Fossa**
Sclerosis (RADIOLOGICAL) vs. Sclerosis (HISTO)	0.47	1.00
Osteophytes (RADIOLOGICAL) vs. Osteophytes (HISTO)	0.62	1.00
SCB lysis/cysts (RADIOLOGICAL) vs. SCB lysis/cysts (HISTO)	0.58	0.24
	**Joint^b^**	
Joint space narrowing (RADIOLOGICAL) vs. Cartilage damage (HISTO)	0.32	

a *Kappa statistic for clustered data. The strength of agreement is generally as follows: ≤ 0.00, poor; 0.01–0.20, slight; 0.21–0.40, fair; 0.41–0.60, moderate; 0.61–0.80, substantial; 0.81–1.00, excellent*.

b *Joint space narrowing (RADIOLOGICAL) was measured for joint. Cartilage damage (HISTO) was measured for Condyle and Fossa separately. If either Condyle or Fossa is “yes,” we define Cartilage damage (HISTO) is “yes” for joint*.

**Table 5:** Summary of association between OA Grade (CBCT) and other grades (side is unadjusted).

		**Outcome**					
		**OA Stage (GROSS)** **(0–4)**		**OA Grade (HISTO) (1–6)**		TOTAL Path OA Score (OA stage ^*^ OA grade)	
**Included samples**	**Factors**	**Odds Ratio** ** (95% CI)**	***P*-value**	**Odds Ratio** ** (95% CI)**	***P*-value**	**Odds Ratio** ** (95% CI)**	***P*-value**
**Head** ^a^	**OA Grade (CBCT)**						
	0/1	Reference	-	Reference	-	Reference	-
	2/3	0.50 (0.12, 2.03)	0.330	5.38 (1.11, 26.02)^*^	0.036	2.54 (0.59, 10.91)	0.211
**Fossa** ^a^	**OA Grade (CBCT)**						
	0/1	Reference	-	Reference	-	Reference	-
	2/3	6.44 (0.97, 42.76)	0.054	1.52 (0.05, 43.17)	0.805	3.28 (0.49, 21.81)	0.219

*Estimated odds ratios on increasing to the next higher level of outcome (other grade) were reported. OR>1 means higher probability to have higher grade*.

* *indicates p < 0.05*.

a *Ordinal logistic mixed-effects models were used, with OA Grade (CBCT) as fixed effects. Random intercept was included in model to account for within-animal correlation whenever necessary*.

#### Excellent Agreement Was Found for Both Sclerosis and Osteophytes Observed on CBCT and Histology on Mandibular Fossas

Considering both joints (right and left), subchondral bone sclerosis observed by CBCT on mandibular fossas correlated perfectly (excellent agreement) with histologic evidence of sclerosis (κ = 1.00) (Table 4; Figure 1). Similarly, excellent agreement was achieved upon evaluation of osteophytes found on CBCT and histologic evaluation of the mandibular fossa (κ = 1.00) (Table 4; Figure 2).

**Figure 5:**
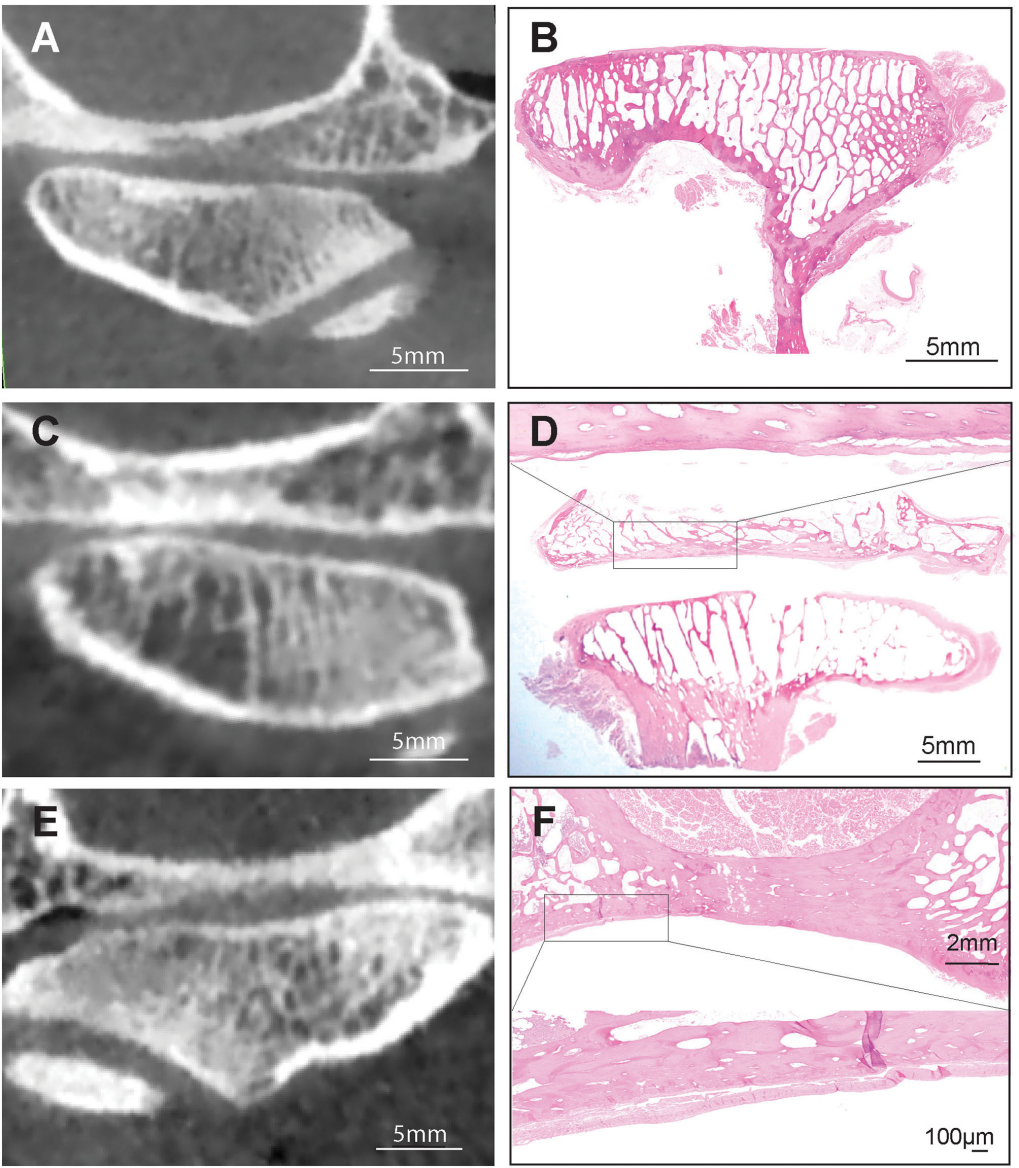
Series of CBCT and histological images demonstrating the fair to moderate agreement between CBCT and histology on subchondral bone sclerosis **(A,B)**, between joint space narrowing and articular cartilage damage **(C,D)** and subchondral bone erosion **(E,F)**. Hematoxilin & Eosin staining, Bar = 5 mm and closeup Bar = 100 μm.

All other measured agreements were characterized as either “fair,” “moderate,” or “substantial” (Table 4). The strength of agreement was not found to be “poor” or “slight” in any instance (κ ≤ 0.20) (Figure 5). Agreement between joint space narrowing on CBCT and cartilage damage on histology was fair (κ = 0.32). Similarly, fair agreement was observed between SCB lysis/cysts found on histologic evaluation and CBCT observation (κ = 0.24). There was moderate agreement between histologic and radiological evidence of both sclerosis (κ = 0.47) and subchondral bone lysis/cysts (κ = 0.58) on the mandibular heads. Additionally, moderate agreement was found between histologic and radiologic findings of SCB lysis/cysts on the mandibular heads (κ = 0.58) (Figure 3). There was substantial agreement on osteophytes of mandibular heads (κ = 0.62) (Table 4).

#### Association Between Gross Pathology OA Grade, Histological OA Stage, and CBCT OA Grade Increases With Greater Severity of Degenerative Changes

In the analysis unadjusted for side, high OA grade (CBCT) was significantly associated with high histological OA stage in the mandibular head (OR = 5.38; *p* = 0.036). The association between gross pathology OA grade and CBCT OA grade in mandibular fossas was near the edge of significance (OR = 6.44, *p* = 0.054) (Table 5).

Adjustment for sides lead to similar results (Supplementary Table 1): high OA grade (CBCT) was significantly associated with high histological OA Stage in the mandibular head (OR = 5.42; *p* = 0.037); the association between gross pathology OA grade and CBCT OA grade in mandibular fossas was near the edge of significance (OR = 6.71, *p* = 0.052).

## Discussion

To our knowledge, this study is the first to evaluate the diagnostic yield of CBCT for diagnosis of degenerative changes of the TMJ in dogs. We demonstrated that subchondral bone sclerosis was the most common finding radiographically and histologically with a fair to excellent agreement. Also, CBCT and histology had moderate to an excellent agreement on the presence of osteophytes. Finally, the more severe the TMJ degenerative changes, the higher was the agreement between CBCT and histology. Taken together, and with few exceptions, CBCT was found to be a suitable modality to evaluate degenerative changes of the TMJ.

Subchondral bone sclerosis is defined as an increase in bone density in the osseous components of the TMJ and has been historically reported to be an indicator of arthritic lesions of the TMJ (7). However, more recently, subchondral bone sclerosis has been recognized as an adaptive change to the forces acting upon the components of the TMJ (7, 31). Repetitive loading of trabecular bone bends the trabecula (strain) and thus acts as a shock absorber to dissipate peak forces away from the joint surfaces. Subchondral sclerosis reduces the trabecular bone strain and allows for more peak forces to concentrate at the joint surface thus increasing the potential for DJD. In addition, subchondral bone sclerosis is one of the first changes that indicate that the functional threshold of biomechanical forces has been exceeded, and thus, resulted in an adaptive response by increasing bone trabeculation (7, 31). Subchondral bone sclerosis was noted previously in people with a reported prevalence in 30.2% of joints followed closely by surface erosion in 29.3% of joints (32). Conversely, in several other studies focusing on the CBCT findings in human patients with TMJ osteoarthritis, sclerosis was not reported as the most common finding (32–34). In the dog, we found that subchondral bone sclerosis was the most common finding on both radiographic and histologic evaluation with fair to excellent agreement. However, subchondral sclerosis was not always associated with the presence of other degenerative changes. It is possible that in the dog's TMJ, the subchondral bone sclerosis may be an early indicator of a degenerative process or, alternatively, may represent a normal aging change. In the present study, age-related subchondral bone sclerosis could not be established as the vast majority of dogs were between the age of 7 and 16-years-old and most had sclerotic changes on at least one joint surface on CBCT and histology. Further studies would be necessary to determine the clinical significance of this finding and if indeed subchondral bone sclerosis occurs as a function of age.

In addition to subchondral bone sclerosis, osteophytes are one of the main radiographic features of degenerative joint disease (7, 33–35). An osteophyte is defined as “a fibrocartilage-capped bony outgrowth” that typically forms in later stages of disease as an adaptive response to joint damage (36, 37). Osteophytes form with the purpose of withstanding loading forces and improve joint stability by increasing the surface area of the joint (36, 37). Clinically, osteophytes may cause discomfort and joint dysfunction, and thus are important to note when evaluating a patient for DTMJ (36, 37). Similar to our findings on subchondral sclerosis, we demonstrated that osteophytes also had moderate to excellent agreement on CBCT and histology. However, the prevalence of osteophyte detection was much lower than of subchondral bone sclerosis. Presence of osteophytes on gross observation was difficult to assert due to small size and potential shape change of the condylar process due to bone remodeling. Hence, confirmation of osteophytes should be ideally done via histology rather than gross observations.

In addition to the aforementioned hard tissue changes associated with DTMJ, disc pathology is also an important feature relevant to clinical decision-making. With regards to the TMJ, disc derangements include fibrillogenesis, cartilage/chondrocyte loss, neovascularization, calcification, and perforation (38). Lastly, synovial changes included inflammation and proliferation of the synovial tissue (38). The criteria denoted in Wilkes staging of TMJ internal derangements serve as a gold standard reference for recognizing the main internal joint changes that are associated with different clinical presentations of human degenerative disease (14, 39). More specifically, disc perforation is cited as a feature of late-stage degenerative disease in humans, and more than half of patients with non-reducing anterolateral displacements develop a perforation in the posterior attachment (14, 39). The observation of joint space narrowing and increase in joint congruency improve the likelihood of detecting disc perforation grossly (7). In that context, we found that bilateral disc perforation naturally occurred in two dogs. Disc perforation has only been cited once previously in the dog, and the prevalence and clinical significance in veterinary patients are currently unknown (3). These findings invite further investigation since in the cases of TMJ disc perforation we report here, no significant degenerative changes were observed in the osseous components of the TMJ.

Finally, we demonstrated that the more severe the degenerative changes, the greater the agreement between CBCT and histology. Several studies have been conducted comparing histological and radiological findings of various diseases in veterinary medicine (19, 21, 25). With regards to osteoarthritis, a positive correlation between magnetic resonance imaging findings and histologic lesions in osteoarthritic hip and knee joints in dogs were documented (19, 20, 22). Similarly, comparison studies have been performed in humans correlating histology to radiology in a variety of osteoarthritic diseases in the hip, knee, and trapeziometacarpal joint revealing positive agreement between the modalities (26, 40). The present study reports that, with few exceptions, a histology/radiology correlation exists with degenerative changes of the TMJ. With regards to disorders of the TMJ specifically, two reports compared data amongst radiological and histological findings in human patients, though neither study used statistics to evaluate the agreement between the two methods (41, 42). This knowledge emphasizes the importance and novelty of the data presented in the present study.

It is important to note that the strength of agreement was fair to moderate for mild to moderate degenerative changes. The disparity between histopathologic and radiographic evidence of osteoarthritis has been previously reported in dogs (43). In a study focused on the effects of food restriction on the development and progression of canine shoulder joint OA, it was found that radiographic evaluation does not represent the severity of joint pathology. Specifically, 40 of 46 dogs (87%), with histopathologic evidence of OA, had a well-defined humeral lesion, which was not diagnosed radiographically (43). Similarly, lack of agreement between the severity of radiographic findings and clinical signs of TMJ pain has been reported in humans (44). The imperfect agreement between CBCT and histopathology in characterizing mild to moderate TMJ degeneration is likely due to two key factors: first, the two diagnostic modalities have inherently different capabilities and CBCT provides limited ability to assess the joint soft tissues, and second, our understanding of the events differentiating physiologic adaptation from early pathology is likely incomplete in the canine TMJ.

The anatomic structures and tissues of the TMJs (articular cartilage and subchondral bone) constantly adapt to stress to optimize their functional capacity. Joint failure or degenerative joint disease (DJD) may occur when the stresses received by tissues exceeds their adaptive capacity. Joint tissue affected by DJD respond by altering their form (size and shape) and structure to reduce the stresses to a level that will promote tissue repair. The process of tissue response, failure and repair occurs in a continuum or predictable sequence that can be monitored with imaging. Humans experiencing the later stages of DJD generally have a significant reduction in the clinical signs and symptoms. In humans the radiographic patterns of adaptation (remodeling), failure (active DJD), and repair (stable DJD) occurs in the following sequence: cortical thickening of the articular surface, mild flattening of the articular surface, cortical erosions, cavitation defects and loss of condylar volume at the erosion sites, extreme flattening of the articular surfaces, formation of subchondral bone cysts, recortication of the articular surface, tendency toward joint congruency and osteophyte formation (30). More studies are needed to define the pathophysiology of TMJ degeneration in the dog as well as the specific aspects that prohibit stronger correlation between CBCT and histopathology. This is important because radiology or imaging is the only tool available to clinicians for antemortem diagnosis of DTMJ.

The limitation of this study is inherent to cadaveric-based studies, which preclude correlation of imaging and histological findings with clinical presentation. However, in our previous study, we demonstrated that these degenerative changes do not correlate with the pain (17). An additional limitation is in the use of an established ICRS/OARSI OA grading system developed for appendicular joints (27). We chose to use this previously validated and universally used system, even if not specific to the TMJ while acknowledging that it may not be entirely clinically applicable as there is no validated scoring system universally accepted for the TMJ. As mentioned earlier, sclerosis was the most common finding in the TMJ. According to the ICRS/OARSI scoring system, sclerosis corresponds to a relatively high grade and stage of OA. With a high score of OA, it would be expected to identify other degenerative changes in addition to sclerosis. However, it was not always the case with canine TMJs. It is possible the OA scoring system needs to be adjusted to the unique features of TMJ joint. Furthermore, temporal studies would be necessary to establish potential age-related changes of the TMJ joint in dogs to prevent potential overinterpretation of the histological and radiographic findings as degenerative.

In conclusion, we evaluated the diagnostic yield of CBCT in the diagnosis of degenerative changes of the TMJ in the dog. We found that the more severe the TMJ degenerative changes, the better the agreement between CBCT and histology. In mild degenerative changes, the correlation is weaker. Additionally, we found that histological evidence of both subchondral bone sclerosis and osteophytes correlated well with CBCT findings, with sclerosis being the most prevalent finding. Finally, we demonstrated that CBCT is a suitable imaging modality to evaluate degenerative changes of the TMJ, but more work is needed before TMJ pathology can be confidently determined with radiologic methods.

## Data Availability Statement

The original contributions presented in the study are included in the article/Supplementary Material, further inquiries can be directed to the corresponding author.

## Ethics Statement

Ethical review and approval was not required for the animal study because it was a cadaveric-based study, IACUC approval was not needed. Written informed consent was obtained from the animal owners for their participation in this study.

## Author Contributions

RM: collection and assembly of data, data analysis, writing of manuscript, and final approval of manuscript. NV and DH: collection and assembly of data, data analysis and interpretation, writing of manuscript, and final approval of manuscript. NC: collection and assembly of data and final approval of manuscript. SC: statistical analysis. FV: review of the manuscript for important intellectual input. DC: conception and design, financial support, collection and assembly of data, data analysis and interpretation, and final approval of the manuscript. BA: conception and design, financial support, provision of study material or patients, manuscript writing, data analysis and interpretation, and final approval of manuscript. All authors contributed to the article and approved the submitted version.

## Conflict of Interest

The authors declare that the research was conducted in the absence of any commercial or financial relationships that could be construed as a potential conflict of interest.

## Publisher's Note

All claims expressed in this article are solely those of the authors and do not necessarily represent those of their affiliated organizations, or those of the publisher, the editors and the reviewers. Any product that may be evaluated in this article, or claim that may be made by its manufacturer, is not guaranteed or endorsed by the publisher.
