# Supplementary material for: The Diagnostic Yield of Cone-Beam Computed Tomography for Degenerative Changes of the Temporomandibular Joint in Dogs
Source: Front Vet Sci. 2021 Aug 4;8:720641. doi: 10.3389/fvets.2021.720641 (PMC8371634; doi:10.3389/fvets.2021.720641)
Supplement: Supplementary file 1 [file Table_1.docx]

**Supplementary Table 1. Summary of association between OA Grade (CBCT) and other grades, adjusted by side and bone. Estimated odds ratios on increasing to the next higher level of outcome (other grade) were reported. OR>1 means higher probability to have higher grade. * indicates p<0.05.**

|  |  | **Outcome** | | | | | |
| --- | --- | --- | --- | --- | --- | --- | --- |
|  |  | **OA Stage (GROSS)**  **(0-4)** | | **OA Grade (HISTO)**  **(1-6)** | | **TOTAL Path OA Score (OA stage * OA grade)** | |
| **Included samples** | **Factors** | **Odds Ratio**  **(95% CI)** | **P-value** | **Odds Ratio**  **(95% CI)** | **P-value** | **Odds Ratio**  **(95% CI)** | **P-value** |
| **Head**^a^ | **OA Grade (CBCT)** |  |  |  |  |  |  |
|  | 0/1 | Reference | - | Reference | - | Reference | - |
|  | 2/3 | 0.53 (0.13, 2.19) | 0.384 | 5.42 (1.11, 26.54)* | 0.037 | 2.57 (0.60, 11.06) | 0.205 |
|  | **Side** |  |  |  |  |  |  |
|  | Right | Reference | - | Reference | - | Reference | - |
|  | Left | 0.65 (0.19, 2.26) | 0.499 | 2.92 (0.73, 11.70) | 0.131 | 0.84 (0.23, 3.01) | 0.789 |
| **Fossae**^e^ | **OA Grade (CBCT)** |  |  |  |  |  |  |
|  | 0/1 | Reference | - | Reference | - | Reference | - |
|  | 2/3 | 6.71 (0.98, 45.80) | 0.052 | 1.44 (0.05, 40.87) | 0.832 | 3.14 (0.47, 21.18) | 0.240 |
|  | **Side** |  |  |  |  |  |  |
|  | Right | Reference | - | Reference | - | Reference | - |
|  | Left | 0.51 (0.13, 1.92) | 0.318 | 0.46 (0.04, 5.70) | 0.544 | 0.56 (0.14, 2.18) | 0.404 |

^a^Ordinal logistic mixed-effects models were used. Fixed effects include OA Grade (CBCT), side, bone, and the interaction between bone and others. The interactions were removed from final models due to non-significance. Random intercept was included in model to account for within-animal correlation whenever necessary.
